# Camera movement impacts on mu-wave activity during action observation in adults with Autism Spectrum Disorders without intellectual disabilities

**DOI:** 10.1192/j.eurpsy.2025.526

**Published:** 2025-08-26

**Authors:** B. Demartini, V. Nisticò, R. Del Giudice, F. Serio, G. Boido, G. Ingrosso, F. Lombardi, C. Sanguineti, V. Casula, A. Baccara, E. Chiudinelli, F. Vairano, F. M. Panzeri, M. Giori, P. M. Inghilleri di Villadauro, R. Faggioli, O. Gambini, T. Subini

**Affiliations:** 1 Dipartimento di Salute Mentale e Dipendenze, ASST Santi Paolo e Carlo; 2 Department of Health Science; 3 Dipartimento di Beni culturali e ambientali, University of Milan, Milan, Italy

## Abstract

**Introduction:**

Despite extensive investigation, the neurophysiological underpinnings of ASD remain poorly understood; a predominant hypothesis relates to the activity of the brain’s mu-rhythm (defined as the frequency band ranging between 8–14 Hz topographically centered over the sensorimotor cortex) and the so-called “Mirror Neuron System” (MNS), considered as an execution-imagination-observation matching system. In particular, mirror neurons (MN) are bimodal neurons located in the ventral premotor cortex, discharging both when a goal-directed action is performed and when it is observed. Previous studies investigating mu-wave suppression in individuals with ASD were limited by having consistently employed video clips filmed with a fixed camera position, hence not including the potential movement of the observer towards the other person (a condition perceived as more ecological and close to reality).

**Objectives:**

Aim of this study was to investigate differences in mu-wave modulation in individuals with Autism Spectrum Disorder (ASD) without intellectual disabilities with respect to a group of neurotypical controls (NT).

**Methods:**

Thirty individuals with ASD and thirty NT underwent an EEG recording while watching short videos depicting goal-oriented action filmed from a fixed position, zooming in on the scene, and approaching the scene by means of a steadycam. Afterwards, participants underwent a rating task to evaluate their subjective viewing experience.

**Results:**

Steadycam videos elicited enhanced event-related desynchronization (ERD), suggestive of an enhanced neural activity, in the NT group, and a reduced ERD in the ASD group, with respect to the other filming conditions. ASD participants also showed difficulties in returning to baseline mu-power levels after watching videos filmed from a fixed position. NT reported to feel more comfortable watching videos with movement, whereas participants with ASD did not exhibit differences between conditions.

**Conclusions:**

The ecological nature of video recording (i.e., visual stimuli filmed with a steadycam) seems to have an opposite effect on the ASD an NT population, as it enhances mu-wave suppression in NT, and reduces it in individuals with ASD, bringing them to a level of desynchronization closer to the one of NT. Understanding these differences might help developing tailored interventions to support perceptual, cognitive, and social processes of people with ASD.

**Disclosure of Interest:**

None Declared

